# Creating a contemporary clerkship curriculum: the flipped classroom model in emergency medicine

**DOI:** 10.1186/s12245-016-0123-6

**Published:** 2016-09-13

**Authors:** Edward K. Lew

**Affiliations:** Department of Emergency Medicine, Stanford University, 300 Pasteur Drive, Alway Building, M121, Stanford, CA 94305-2200 USA

**Keywords:** Flipped classroom, Student, Clerkship, Online, Fourth year, Curriculum

## Abstract

**Background:**

The teaching modality of “flipping the classroom” has garnered recent attention in medical education. In this model, the lecture and homework components are reversed. The flipped classroom lends itself to more interaction in “class” and theoretically improved clinical decision-making. Data is lacking for this model for students in emergency medicine clerkships. We trialed the flipped classroom in our fourth-year student clerkship. Our aim was to learn student and faculty facilitator perceptions of the experience, as it has not been done previously in this setting. We evaluated this in two ways: (1) participant perception of the experience and (2) facilitator (EM physician educator) perception of student preparation, participation, and knowledge synthesis.

**Methods:**

With permission from its creators, we utilized an online video series derived from the Clerkship Directors in Emergency Medicine. Students were provided the link to these 1 week prior to the classroom experience as the “homework.” We developed patient cases generated from the videos that we discussed during class in small-group format. Afterward, students were surveyed about the experience using four-point Likert items and free-text comments and also were evaluated by the facilitator on a nine-point scale.

**Results:**

Forty-six clerkship students participated. Students deemed the online modules useful at 2.9 (95 % CI 2.7–3.2). Further, they reported the in-class discussion to be of high value at 3.9 (95 % CI 3.8–4.0), much preferred the flipped classroom to traditional lecturing at 3.8 (95 % CI 3.6–3.9), and rated the overall experience highly at 3.8 (95 % CI 3.7–3.9). Based on preparation, participation, and knowledge synthesis, the facilitator judged participants favorably at 7.4 (95 % CI 7.0–7.8). Students commented that the interactivity, discussion, and medical decision-making were advantages of this format.

**Conclusions:**

Students found high value in the flipped classroom and prefer it to traditional lecturing, citing interactivity and discussion as the main reasons. The facilitator also viewed that the students were not only well prepared for the flipped classroom but that they also actively participated in and synthesized knowledge adequately during this experience. This study supports the use of the flipped classroom for EM clerkship students as a valuable, preferable teaching technique.

**Electronic supplementary material:**

The online version of this article (doi:10.1186/s12245-016-0123-6) contains supplementary material, which is available to authorized users.

## Background

The flipped classroom modality has garnered international attention in recent years as an alternative to traditional teaching in medical education [1–6]. Subjects utilizing it range from OB-GYN to palliative care to anesthesia to emergency medicine (EM) [1, 2, 4–6]. In this method, the roles of traditional lectures “in-class” and homework are reversed. Learners are generally assigned content to review at home prior to classroom instruction. Many times, this is done asynchronously through video lectures or interactive modules online. Classroom time is then used to apply that knowledge by active learning, working through problems where there is an expert to clarify concepts. Benefits of the asynchronous portion include learners able to access and complete a module on the learner’s own time and pace. They may pause, rewind, or speed through any portion of the asynchronous component. It is learner-led. In regard to the classroom portion, it has been suggested that learners benefit from group interaction with each other and with the expert educator to guide knowledge application—giving instant feedback [7–9]. This technique has been argued to be ideal for medical education by building a framework of core knowledge in the pre-class assignments and then reinforcing and embedding that knowledge through an interactive format [3].

As a specialty, EM is beginning to use this technique as a teaching method. Mainly, this has been in the post-graduate years [4, 6]. Thus far, little existing research has been published in this field for EM students. A recent survey of EM clerkship directors showed that more than half of US medical schools are requiring rotations in EM [10]. As schools move toward competency-based models [10], it will be increasingly vital to explore different teaching techniques other than traditional lecturing.

We instituted the flipped classroom into our EM clerkship as an additional avenue to better teach our students, promote active learning, and innovate the curriculum. Our aim was to learn both student and faculty facilitator perceptions of this experience. To our knowledge, this is the first description of the flipped classroom in the EM clerkship.

## Methods

### Study setting and population

The study site was a tertiary-care, academic hospital that is home to a US Accreditation Council for Graduate Medical Education (ACGME)-accredited Emergency Medicine Residency program and an elective fourth-year medical student clerkship. Different fourth-year medical students rotate monthly, ranging from those interested in pursuing EM residency to those rotating to complete school credits or out of pure interest. Much less frequently, third-year medical students and physician-assistant students also took the clerkship. All students rotating in the clerkship were eligible. This study was exempt by our Institutional Review Board.

### Study flipped classroom curriculum

With permission from its creators to use in this study, we utilized online video portions of the website http://flippedemclassroom.wordpress.com. This site, which is part of the FOAM (Free Open Access Medical Education) movement, derived its modules from the student curriculum endorsed by the Clerkship Directors in Emergency Medicine (CDEM) [11, 12]. In particular, we used the “Approach to Chest Pain,” for our flipped classroom model. From these modules, we developed patient cases ranging from acute coronary syndrome to aortic dissection to pulmonary embolism. Microsoft PowerPoint slides were created to present imaging (such as chest x-rays), EKGs, and lab values, during the class. Scripts for the patient scenarios were also provided to the facilitator, who led all the sessions throughout the year. The facilitator was an Emergency Medicine Medical Education Fellowship-trained faculty member.

One week prior to the in-class session, the students were sent an email with links to the specific videos on chest pain. The email also detailed the necessity to view the modules prior to class, in order to make the session more interactive and useful to their learning. The in-class sessions were small-group discussion format with the facilitator guiding the discussion using the patient cases and slides. It was noted to the students that this session would have no bearing on their final clerkship grades.

Afterward, the facilitator evaluated them individually based on participation, preparation, and knowledge—3 points for each, for a total of 9. This grading rubric was in line with what was used at our institution (though this session was not officially part of the students’ final grades). The students were also surveyed about the experience via paper survey utilizing a four-point Likert scale querying four items: usefulness of overall experience, the online portion, the classroom portion, and preference of flipped classroom format versus traditional lectures (Additional file 1). Students were also specifically asked what they liked about the flipped format, what they did not like, and also ways to improve the experience. These were written free-text responses. The free-text responses were then reviewed and grouped into three separate themes based on similarities.

## Results

During the 1-year study period (August 2014–August 2015), 46 students were enrolled and participated in the new clerkship flipped classroom experience with a mean class size of 5 students. All of them answered the non-required survey. On a nine-point scale based on participation, preparation, and knowledge, the facilitator rated each student and found the students to do well in those aspects with a mean score of 7.4 (95 % CI 7.0–7.8).

Student survey results (four-point Likert scale) revealed that they found the overall experience to be extremely useful with a score of 3.8 (95 % CI 3.7–3.9), online portion useful with a score of 2.9 (95 % CI 2.7–3.2), interactive cases extremely useful with a score of 3.9 (95 % CI 3.8–4.0), and much preferred flipped versus traditional with a score of 3.8 (95 % CI 3.6–3.9).

Of the three determined themes that students liked about the flipped classroom, (1) 54.3 % responded to enjoying the high level of involvement, interactivity, and/or discussion; (2) 26.1 % responded to liking the critical thinking and medical decision-making; and (3) 19.6 % responded to specifically enjoying the flipped classroom format (i.e., online homework than in-class case-based scenarios) (Table 1).

**Table 1:** Representative student responses to open-ended questions of flipped classroom survey

What did you like about this format?	What didn’t you like about this format?
Interactive. Case presentations we worked through	Students competing to get thoughts in can be distracting.
It was presented like how we would see patients in the ED and it emphasized the not-so-clear decision patients that can be encountered	Online videos were useful review but did not go in-depth for new learning
Interactive; good thinking process for tough clinical situations	The discussion is a bit of a free-for-all. For those who do not like talking over others, this is a challenging format
I felt that it was much easier to learn actively instead of passively	Seems like we hit the major learning points, but not sure - variable experience depending on group
High quality chalk-talk style lectures, and then the in-class interactive portion allows us to apply & solidify what I just learned	It’s harder for everyone to be able to participate equally especially if you are a quiet person
Puts in mindset of clinical approach. Helps clarify factors going into decision-making	Sometimes I know the answers and you have to deal with others putting out weird recommendations
The case application and discussion following the online learning/review	I wish the videos were a little more advanced, much of the information I already felt familiar with
Learning from others is more effective and engaging than lecture; there is more freedom/comfort to ask questions	A little slower than traditional content
Spending my own time learning the basics and having time to apply the concepts and info	Hard to follow everything we’d done in each case at times. Pre-classroom work needed to be more advanced
Closer to real decision-making, time to discuss/debate/clarify knowledge and misconceptions	Sometimes a little rushed with more advanced topics, but felt comfortable checking in/asking questions

Of the three determined themes that students disliked about the flipped classroom, (1) 30.4 % mentioned difficulties in the small-group format; (2) 26.1 % mentioned the videos; and (3) 43.5 % did not respond or did not answer the question (e.g., “I liked everything about it”) (Table 1).

When asked to free-text respond how to improve the experience, 50 % of learners either left the field blank, wrote that nothing could be improved, or wrote something not directly related to improving the flipped classroom experience (e.g., “Nothing, thought it was well done”). 21.7 % suggested expanding this method into other chief complaints or including many more cases. 19.6 % thought the online videos could be improved ranging from depth to length of time. 8.7 % thought the interactive portion would benefit from better moderation.

## Discussion

The flipped classroom concept provides valuable medical education opportunities for its learners. There have been calls for its increased use in medical education [3]. Although increasingly becoming more prevalent in medicine, to our knowledge, this is one of the first studies evaluating this modality at the EM undergraduate clerkship level. Our successful implementation reveals that clerkship students not only highly valued the approach but also preferred it to traditional lecture format. Further, facilitator evaluation of the students was favorable as well.

As the survey revealed, there was overall high utility in the flipped experience and preference over traditional lecturing. Comments augmented this as a majority enjoyed the interactivity and discussion during the case-based classroom portion. Additionally, a quarter of students cited the medical decision-making process as important to them. This reflects previous study findings at the graduate medical education level [4, 6]. It is theorized that in adult learners, this setup promotes synthesis, analysis, and direct application of knowledge [4, 13, 14], which seem to be what our students valued the most. This falls in line with the idea that we can improve clinical reasoning by providing immediate feedback in our discussion of learner judgment and reasoning [8]. The small-group format is ideal for this.

One of the major criticisms of the flipped classroom is preparation. If learners do not complete the “homework” beforehand, it can be difficult to have a successful classroom portion. In addition, even if they did the “homework,” opponents argue that some students would not benefit from in-class discussion based on not interacting. However, our study refutes both those claims and shows that for the clerkship students, the facilitator found the participants to be well prepared, knowledgeable, and participatory. The learners also support this as they rated the interactive case portion as the most useful aspect (3.9/4.0). Given that this session did not factor into their clerkship grades, we further believe our learners were not motivated in that regard.

As is seen in other specialties, once implemented, students would like to see more of the flipped pedagogy in more aspects of their education [1]. This was similar to our experience. In employing the “Approach to Chest Pain,” more than a fifth of learners admitted a desire to implement this modality for other EM chief complaints during their EM curriculum. When asked specifically, 50 % thought our model did not need immediate improvement. Of those that did comment about improvement, many thought the video portion could be better. It is important to note that we used a previously made online module that was derived from CDEM, which created learning objectives for all students (not just those who are pursuing EM residency). Most thought that the videos could be more comprehensive, but this may be because our clerkship was elective; therefore, many students were likely EM-bound, seeking to learn more extensive EM knowledge. The flipped classroom ideally may benefit from its facilitators creating both the “homework” and in-class portions.

Given its success in the chest pain module, the next steps would be to expand to additional patient complaints. Possibly, the inclusion of simulation may provide even more real-life interactivity and “on-the-spot” training in a low-pressure environment. These results are promising for the EM clerkship and bolster the case for case-based learning, specifically using the flipped classroom approach. In the flipped classroom, learners assimilate core knowledge at their own leisure, educators save time from repetitive activities, and the setup promotes higher-order interactive knowledge sharing [4, 15–17].

This study took place at a single institution that is an academic hospital. Students are not required to take the EM clerkship, and as such, more learners were likely EM-bound—possibly more motivated. However, students were explicitly notified that this flipped classroom would not affect their grades. Results may not be generalizable to other types of institutions and clerkships. Not being a required clerkship also led to an overall small sample size. We hoped to alleviate this analytical issue with the free comment section, and it appears that free comments helped to capture the full range of attitudes and perceptions.

A single facilitator was used and may not be realistic in an expanded curriculum needing increased faculty time. Nonetheless, this negated any inter-operator variability and provided a more accurate assessment of the students. Notably, the facilitator was also fellowship-trained in medical education; thus, additional training is likely needed for those who would like to facilitate.

We recognize the assessments made from students and the facilitator to be more subjective in nature, and our approach did not directly compare other teaching modalities. However, given that the flipped approach was new to the clerkship, and had not been reported on for EM clerkship learners before, we thought it reasonable to first seek perceptions and preferences from both students and the facilitator, as this has been done in other specialties [1]. As suggested by this study’s comments, the next step would be expanding the flipped classroom experience. This would naturally be an important future step along with evaluating retention and directly comparing to other teaching modalities.

## Conclusions

Our EM clerkship students found high value in our version of the flipped classroom, appreciated the interactive case-based format, and preferred it to traditional lecturing. The facilitator also viewed that the students were not only well prepared for the flipped classroom but that they also actively participated in and synthesized knowledge adequately during this experience. This study supports the use of the flipped classroom for EM clerkship students as a valuable, preferable teaching technique. In sync with learner comments, future steps include expansion of this approach and evaluation of retention and comparison to other teaching modalities.

## Additional file

Additional file 1: Flipped EM classroom for fourth-year medical students survey. (DOCX 19 kb)

## Abbreviations

EM: Emergency medicine
CI: Confidence interval
CDEM: Clerkship Directors in Emergency Medicine
