# Supplementary material for: Creating a contemporary clerkship curriculum: the flipped classroom model in emergency medicine
Source: Int J Emerg Med. 2016 Sep 13;9(1):25. doi: 10.1186/s12245-016-0123-6 (PMC5021642; doi:10.1186/s12245-016-0123-6)
Supplement: Additional file 1: — Flipped EM classroom for fourth-year medical students survey. (DOCX 19 kb) [file 12245_2016_123_MOESM1_ESM.docx]

**Flipped EM classroom for Fourth-year medical students survey**

***1) How useful to your learning did you find the overall flipped EM classroom format?***

| Not useful at all |  |  | Extremely useful |
| --- | --- | --- | --- |
| 1 | 2 | 3 | 4 |

***2) How useful to your learning did you find the online portion of the flipped EM classroom?***

| Did not view | Not useful at all |  |  | Extremely useful |
| --- | --- | --- | --- | --- |
| N/A | 1 | 2 | 3 | 4 |

***3) How useful to your learning did you find the interactive (classroom) portion?***

| Not useful at all |  |  | Extremely useful |
| --- | --- | --- | --- |
| 1 | 2 | 3 | 4 |

***4) As compared to traditional lecture, how much do you prefer this flipped EM classroom or do you?***

| Do not prefer |  |  | Prefer |
| --- | --- | --- | --- |
| 1 | 2 | 3 | 4 |

**5) What did you like about this format? Please write legibly.**

**6) What didn’t you like about this format? Please write legibly.**

**7) How can we improve on this experience? Please write legibly.**
